# Clinical Contexts of Detection of Benign Transient Hyperphosphatasemia in Infants and Young Children: A Single-Center Retrospective Study

**DOI:** 10.7759/cureus.106496

**Published:** 2026-04-05

**Authors:** Koji Yokoyama, Mitsukazu Mamada

**Affiliations:** 1 Department of Pediatrics, Japanese Red Cross Wakayama Medical Center, Wakayama, JPN

**Keywords:** alkaline phosphatase elevation, incidental detection, infants and toddlers, pediatric laboratory abnormalities, transient hyperphosphatasemia

## Abstract

Background: Transient hyperphosphatasemia (TH) is a benign, self-limited condition characterized by a marked but temporary elevation of serum alkaline phosphatase (ALP), typically exceeding 3-5 times the upper limit of the age-adjusted reference range, in otherwise healthy infants and young children. Because extreme ALP elevations may raise concern for liver or bone disease, clarification of its clinical characteristics is important.

Methods: We conducted a retrospective single-center study of children diagnosed with TH at a tertiary pediatric center. TH was defined as a marked but temporary elevation of serum ALP in the absence of clinical or laboratory evidence of bone or liver disease, with spontaneous normalization within several months. Demographic characteristics, clinical symptoms, associated infections, and laboratory findings (including ALP isoenzyme levels) were analyzed.

Results: Forty-one children were included, with a median age of 13 months (IQR 11-18 months). Associated infections were identified in 29 patients, most commonly viral gastroenteritis and respiratory viral infections, although no single pathogen predominated. In 12 patients, TH was detected incidentally during blood testing performed for unrelated clinical reasons. The median peak ALP level was 2,304 U/L (IQR 1,699-3,985 U/L), with values exceeding 10,000 U/L in some cases. ALP isoenzyme analysis demonstrated elevation of both liver and bone isoenzymes in all patients. Peak ALP levels did not differ between sexes or between patients with and without associated infections.

Conclusions: TH predominantly affects infants and toddlers and may be detected both during evaluation of mild infections and incidentally during routine laboratory testing. Recognition of this benign condition is important to prevent unnecessary diagnostic investigations and to reassure caregivers when marked ALP elevation is encountered in otherwise healthy young children.

## Introduction

Marked elevation of serum alkaline phosphatase (ALP) in infants and young children often raises concern for underlying liver or bone disease, such as rickets, cholestatic liver disease, or other metabolic bone disorders, in clinical practice. However, benign transient hyperphosphatasemia (TH) is a self-limited biochemical condition characterized by marked but temporary elevations of ALP in otherwise healthy infants and young children. Serum ALP levels typically normalize spontaneously within a few months without specific treatment [1,2]. Since its first description, TH has been increasingly recognized in pediatric practice and is often identified incidentally during evaluation for unrelated illnesses.

Despite its benign nature, extreme elevations of ALP may raise concern for serious conditions such as cholestatic liver disease, rickets, or other metabolic bone disorders. Consequently, children with TH may undergo extensive laboratory testing, imaging studies, and specialist consultations, which can cause unnecessary anxiety for caregivers and increase healthcare utilization.

Previous studies have suggested that TH occurs predominantly in early childhood and is frequently detected in association with intercurrent infections. However, the reported clinical characteristics, symptom profiles, and patterns of ALP elevation vary among studies, and data from routine clinical practice remain limited.

The aim of this study was to characterize the demographic features, clinical presentation, laboratory findings, and clinical course of children with TH, and to describe the clinical contexts in which the condition is identified in routine pediatric practice. The primary objective of this study was to characterize the clinical features of TH, while a secondary objective was to compare the clinical contexts of detection between infection-associated and incidentally identified cases.

## Materials and methods

Definition of TH

TH was defined as a condition observed in infants and young children, characterized by a marked (typically >3-5 times the upper limit of the age-adjusted reference range) but temporary elevation of ALP in the absence of clinical or laboratory evidence of bone or liver disease.

Diagnostic features included presentation before five years of age, normal liver function tests and parameters of mineral metabolism, and elevation of both bone and liver ALP isoenzymes. Spontaneous normalization of serum ALP levels within several months was used as a confirmatory criterion during follow-up rather than as an initial inclusion criterion [1,2].

Study design and patient selection

This was a retrospective single-center study conducted at a tertiary pediatric center. We reviewed the electronic medical records of children evaluated between January 2019 and April 2025. Patients were identified by screening for elevated serum ALP levels (typically >3-5 times the upper limit of the age-adjusted reference range), followed by confirmation of a clinical diagnosis of TH. All consecutive cases meeting the predefined diagnostic criteria were included. Eligibility was determined based on clinical and laboratory findings in the absence of underlying liver or bone disease. Follow-up data were reviewed to confirm spontaneous normalization of ALP levels.

Data collection and laboratory evaluation

Clinical data, including age, sex, presenting symptoms, and clinical context at the time of blood sampling, were extracted from medical records. Information on associated infections was obtained based on clinical diagnosis documented in the medical records and, when available, virological testing performed at the discretion of the treating physician. Infections were classified according to the primary clinical diagnosis at the time of presentation.

ALP levels were measured using the standardized method of the International Federation of Clinical Chemistry and Laboratory Medicine (IFCC) at the institutional laboratory [3]. Age-adjusted reference ranges provided by the institutional laboratory were used for interpretation, with upper limits varying by age (approximately 350-500 U/L in infants and young children).

Peak ALP levels were defined as the highest recorded value during the clinical course. ALP isoenzyme analysis was performed using standard electrophoretic methods, and the relative contributions of liver (ALP2) and bone (ALP3) isoenzymes were evaluated.

Follow-up and outcome assessment

Follow-up data were obtained from medical records. Normalization of ALP was defined as a return to age-adjusted reference ranges within three to four months without specific treatment. Clinical outcomes and additional laboratory parameters related to bone and mineral metabolism were also reviewed, where available.

Statistical analysis

Statistical analyses were performed using R Statistical Software (R version 4.5.2; 2025, R Core Team, Vienna, Austria). Continuous variables are presented as median with interquartile range (IQR). Group comparisons were performed using the Mann-Whitney U test, given the non-normal distribution of ALP values.

Ethics statement

The Ethics Committee of the Japanese Red Cross Wakayama Medical Center approved this retrospective study (approval number: 1663) and waived the requirement for individual informed consent.

## Results

Patient characteristics

A total of 41 children with TH were included in this retrospective study. The median age at presentation was 13 months (interquartile range (IQR), 11-18 months). Seventeen patients (41.5%) were boys, and 24 (58.5%) were girls. The median age at initial presentation was 12 months (IQR, 11-13) in males and 14 months (IQR, 11.5-18) in females, consistent with the known epidemiology of TH, which predominantly affects infants and young children. Among the 41 children with available symptom data, fever was present in 23 patients (56.1%), respiratory symptoms in 27 (65.9%), and gastrointestinal symptoms in eight (19.5%) (Table 1).

**Table 1:** Baseline characteristics of children with transient hyperphosphatasemia Baseline demographic and clinical characteristics of the 41 children included in this study. Age is presented as median with interquartile range (IQR). Data are shown as number (percentage) unless otherwise indicated.

Variable	Value
Number of patients	41
Age at presentation, months, median (IQR)	13 (11-18)
Male sex, n (%)	17 (41.5)
Female sex, n (%)	24 (58.5)
Fever, n (%)	23/41 (56.1)
Respiratory symptoms, n (%)	27/41 (65.9)
Gastrointestinal symptoms, n (%)	8/41 (19.5)

Associated infections and incidental detection

An associated infection was identified in a subset of patients (n = 29). These patients presented for medical evaluation because of acute infectious symptoms, such as fever, respiratory symptoms, or gastrointestinal illness, and blood tests performed during the clinical evaluation revealed elevated ALP levels.

Viral gastroenteritis was the most commonly identified infection (n = 4), followed by respiratory syncytial virus (RSV) infection (n = 3), human parainfluenza virus (HPIV) type 3 infection (n = 2), HPIV type 1 infection (n = 1), rhinovirus infection (n = 1), adenovirus infection (n = 1), and influenza A infection (n = 1). Identified viral pathogens accounted for 13 of the 29 infection-associated cases, while the remaining patients were diagnosed based on clinical features without specific pathogen identification. No single viral pathogen predominated (Table 2). 

**Table 2:** Associated infections in patients with transient hyperphosphatasemia (n = 29) Infectious conditions identified in a subset of patients at presentation or during clinical evaluation. Data are shown as number of patients. No single viral pathogen predominated.

Infection type	Number of patients
Viral gastroenteritis	4
Respiratory syncytial virus infection	3
Human parainfluenza virus type 3	2
Human parainfluenza virus type 1	1
Rhinovirus infection	1
Adenovirus infection	1
Influenza A infection	1
Total	29

These infections were the primary reasons for presentation, and ALP elevation was identified during blood testing performed as part of the clinical evaluation.

In patients without evidence of intercurrent infection (n = 12), TH was incidentally detected during blood testing performed for other clinical reasons. These included evaluation for allergic conditions (n = 6), poor appetite or poor weight gain (n = 2), routine follow-up in extremely low birth weight infants (n = 2), surveillance for anemia (n = 1), and routine monitoring in a patient receiving antiarrhythmic medication (n = 1) (Table 3).

**Table 3:** Clinical indications for blood testing in incidentally detected cases without associated infection (n = 12) This table summarizes the clinical reasons for blood testing in patients without evidence of intercurrent infection, in whom transient hyperphosphatasemia was incidentally identified. Data are presented as number of patients.

Indication for blood testing	Number of patients
Evaluation for allergic conditions	6
Poor appetite or poor weight gain	2
Routine follow-up in extremely low birth weight infants	2
Surveillance for anemia	1
Routine monitoring during antiarrhythmic therapy	1
Total	12

These findings indicate that TH may be identified either during evaluation of acute infections or incidentally during blood testing performed for unrelated clinical reasons.

Laboratory findings

**Figure 1:**
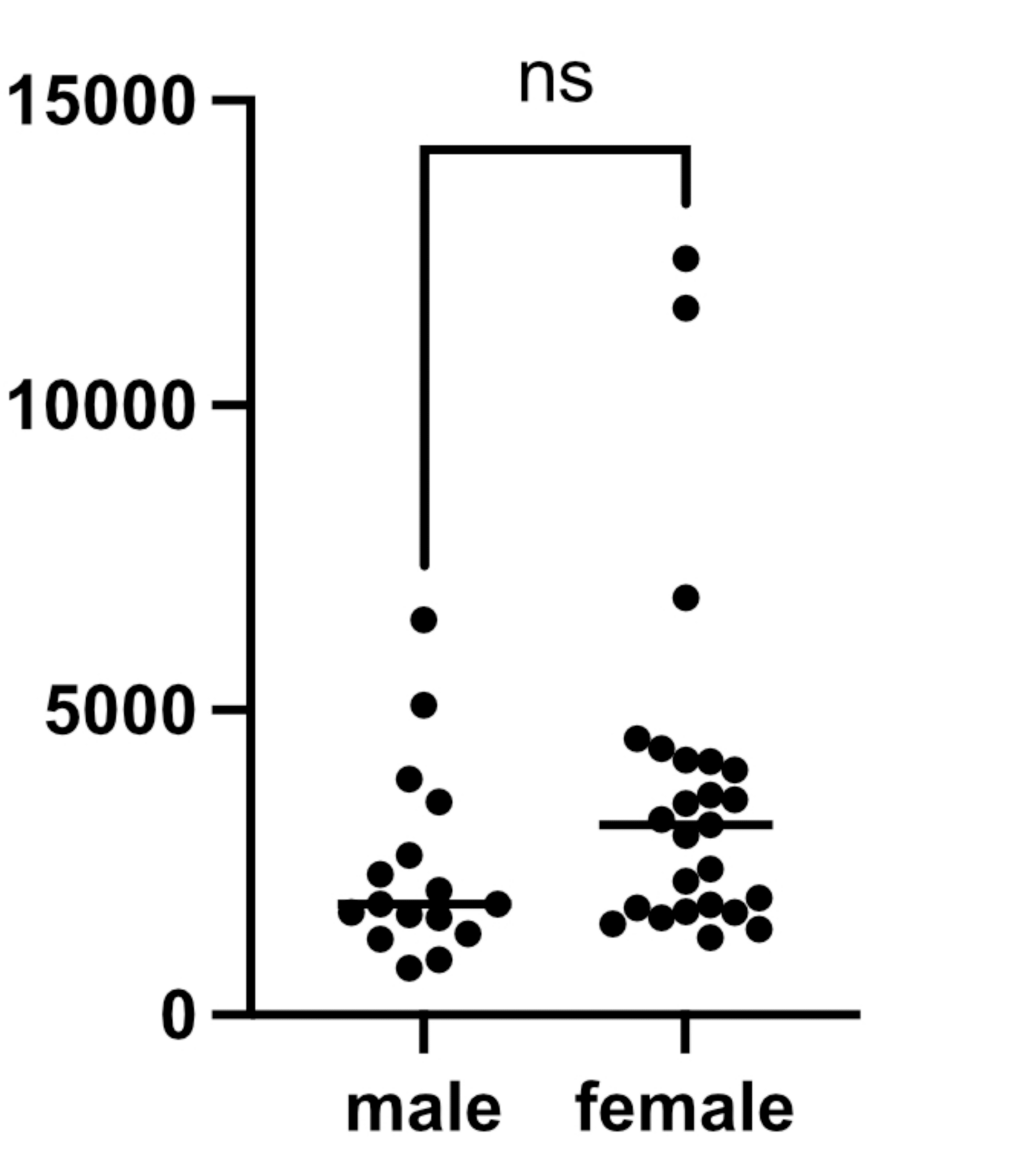
Comparison of peak serum alkaline phosphatase levels between boys and girls with transient hyperphosphatasemia (TH) Peak serum alkaline phosphatase (ALP) levels in boys and girls with TH. The x-axis indicates sex and the y-axis indicates peak serum ALP levels (U/L). Each dot represents an individual patient. No significant difference in peak ALP levels was observed between the two groups (Mann-Whitney U test, p = 0.0839).

**Figure 2:**
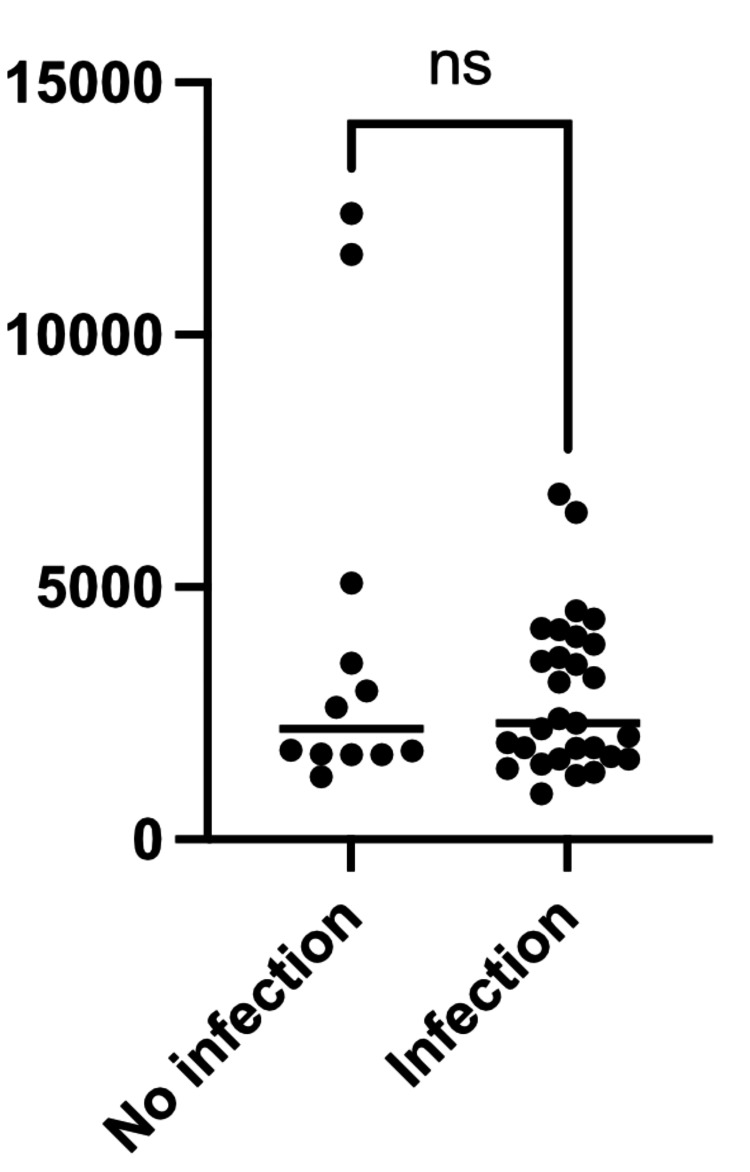
Comparison of peak serum alkaline phosphatase levels between patients with and without associated infections Peak serum alkaline phosphatase (ALP) levels in patients with transient hyperphosphatasemia with associated infections and those without associated infections. The x-axis indicates infection status, and the y-axis indicates peak serum ALP levels (U/L). Each dot represents an individual patient. No significant difference in peak ALP levels was observed between the two groups (Mann-Whitney U test, p = 0.3441).

A total of 115 ALP measurements were analyzed. The mean peak ALP level was 3,153 U/L, with a median of 2,304 U/L (IQR, 1,699-3,985 U/L; range, 900-12,405 U/L). There were no significant differences in peak ALP levels between boys and girls (Figure 1), nor between patients with and without associated infections (Figure 2 and Table 4).

**Table 4:** Laboratory findings and clinical course of children with transient hyperphosphatasemia This table summarizes laboratory characteristics and follow-up findings in children with transient hyperphosphatasemia. Continuous variables are presented as median with interquartile range (IQR) or range, as appropriate.

Variable	Value
Total ALP measurements, n	115
Peak ALP per patient, U/L, median (IQR)	2,304 (1,699-3,985)
Peak ALP range, U/L	900-12,405
Serial decline of ALP during follow-up	Observed in all followed patients
Evidence of liver disease	None
Evidence of metabolic bone disease or rickets	None
Serum calcium, phosphate, iPTH, and 25-hydroxyvitamin D	Within or near age-adjusted reference ranges in most patients

Serial measurements demonstrated a gradual decline in ALP levels in all patients who underwent follow-up testing, without any specific therapeutic intervention. In patients with available follow-up data, serum ALP levels returned to the age-adjusted reference range within several months in all cases, consistent with the expected course of TH. No patient developed clinical or laboratory evidence of underlying liver disease or metabolic bone disease during follow-up.

ALP isoenzyme analysis was performed in all patients and demonstrated a characteristic pattern, with elevation of both liver (ALP2) and bone (ALP3) isoenzymes.

Serum calcium, phosphate, intact parathyroid hormone, and 25-hydroxyvitamin D levels were within or near age-adjusted reference ranges in most patients, and no findings suggestive of rickets were identified.

## Discussion

In this single-center retrospective study, we characterized the clinical features and laboratory course of TH in a cohort of infants and young children. Consistent with previously established diagnostic frameworks, including those described in prior studies and reviews such as Chu and Rothschild [2], TH in our cohort occurred predominantly in early childhood, was not associated with underlying bone or liver disease, and showed spontaneous normalization of serum ALP levels without specific intervention [1,2].

ALP isoenzyme analysis in all patients demonstrated elevation of both liver (ALP2) and bone (ALP3) isoenzymes, consistent with previously reported patterns of TH. Importantly, ALP isoenzyme analysis was available in all patients in our cohort, providing biochemical confirmation of the characteristic elevation of both liver and bone isoenzymes and supporting the diagnosis of TH. These findings reinforce the self-limited nature of this condition [4]. The reported incidence of TH varies across studies, but it is considered an underrecognized condition in routine pediatric practice, particularly in infants and toddlers undergoing laboratory testing for unrelated conditions [5].

One notable observation in our cohort was the variety of clinical contexts in which TH was detected. In many patients, TH was identified during evaluation for mild infectious illnesses, including viral gastroenteritis, RSV infection, and other respiratory viral infections. However, no single viral pathogen predominated. This finding is consistent with previous reports suggesting that TH is not linked to a specific infectious agent but may occur in association with a broad range of infectious or inflammatory conditions [5-7].

Importantly, our results also showed that a substantial proportion of cases were detected incidentally during blood testing performed for unrelated clinical indications, such as evaluation of allergic diseases, growth concerns, or routine follow-up of premature infants. These findings highlight that TH may be discovered both during acute illness and during routine laboratory testing in otherwise clinically stable children. This observation may partly explain why TH is frequently encountered in general pediatric practice despite its relatively low reported prevalence.

The coexistence of infection-associated and incidentally detected cases suggests that the relationship between infection and TH may be complex. In some cases, transient alterations in ALP metabolism may occur in response to inflammatory or infectious stimuli. In other situations, TH may simply be detected because blood tests are more frequently performed during episodes of illness. Our findings therefore support the concept that infection may serve as a clinical context in which TH is detected rather than as a specific causal trigger. The underlying mechanism of TH remains unclear; however, proposed explanations include transient alterations in ALP clearance and changes in circulating ALP isoenzyme properties [1,8]. The degree of ALP elevation observed in our cohort was occasionally striking, with peak values exceeding 10,000 U/L in some patients. Despite these marked biochemical abnormalities, all patients demonstrated spontaneous normalization of ALP levels without clinical sequelae. This observation reinforces previous reports indicating that even extreme ALP elevations can occur in TH and should not necessarily prompt extensive diagnostic evaluation in otherwise well-appearing young children; careful clinical assessment with follow-up may be sufficient in many cases [9,10]. Importantly, the degree of ALP elevation does not correlate with disease severity or prognosis in TH.

Taken together, these findings support the concept that TH represents a benign biochemical phenomenon reflecting temporary alterations in alkaline phosphatase metabolism rather than a manifestation of underlying hepatic or skeletal disease. Recognition of TH is clinically important, as misinterpretation of marked ALP elevation may lead to unnecessary investigations, including imaging and specialist referrals, thereby increasing healthcare burden and parental anxiety. In such situations, TH should be considered in the absence of clinical or laboratory evidence of liver or bone disease [9]. Also, a “wait-and-see” approach with clinical observation and repeat ALP measurement is generally appropriate [8,9]. Awareness of this benign condition may help clinicians avoid unnecessary diagnostic investigations and reduce anxiety among caregivers.

Several limitations should be acknowledged. Because spontaneous normalization was used as a confirmatory criterion, our cohort may have preferentially included cases with a typical benign course, potentially introducing selection bias. First, this study was retrospective and conducted at a single tertiary center, which may limit the generalizability of the findings and introduce selection or referral bias, potentially influencing the clinical spectrum of included cases. In addition, associated infections were identified based on clinical diagnoses and non-standardized virological testing at the discretion of treating physicians, which may have resulted in misclassification or underdetection of infectious etiologies, as virological testing was not uniformly performed across all patients. Furthermore, no adjustment for potential confounders, such as age, was performed due to the limited sample size, which may have influenced the observed associations and should be considered when interpreting the results. In addition, the relatively small sample size likely limited the statistical power to detect differences between subgroups. Therefore, non-significant findings should be interpreted with caution, and the absence of statistically significant differences should not be interpreted as evidence of no association. The timing and frequency of follow-up laboratory assessments were not standardized because testing was performed as part of routine clinical care. Despite these limitations, our findings provide practical clinical insights into the interpretation of marked ALP elevation in infants and young children and may help clinicians recognize TH and avoid unnecessary diagnostic investigations. These findings should be interpreted within the context of a retrospective, descriptive study design.

## Conclusions

TH predominantly affects infants and toddlers and may be detected during evaluation of mild infections or incidentally during routine laboratory testing. It follows a benign, self-limited course even when ALP levels are markedly elevated. Recognition of this condition can help avoid unnecessary investigations and reassure caregivers.
